# Ethambutol-Induced Optic Neuropathy: A Retrospective Study of Incidences and Risk Factors

**DOI:** 10.7759/cureus.79160

**Published:** 2025-02-17

**Authors:** Priti Singh, Samendra Karkhur, Vidhya Verma

**Affiliations:** 1 Ophthalmology, All India Institute of Medical Sciences, Bhopal, Bhopal, IND

**Keywords:** ethambutol, ethambutol-induced optic neuropathy (eon), revised national tuberculosis control programme (rntcp), rnfl thickness, tuberculosis

## Abstract

Purpose: The present study aims to determine the incidences of ethambutol-induced optic neuropathy (EON) and its associated risk factors in Central Indian tertiary care centers.

Materials and methods: A single-centered retrospective observational study was conducted. Data was collected from the Directly Observed Therapy Short-course (DOTS) centre after the revised National Tuberculosis Control Program (RNTCP) 2016 guidelines were implemented. The collected data included demographic data, history of underlying diseases, such as diabetes mellitus, hypertension, HIV infection, and history of smoking. The site of tuberculosis infection, daily dose, the duration of ethambutol (EMB) treatment, onset, and type of ocular symptoms were recorded. We performed regression analyses to investigate the univariate and multivariable associations with different variables.

Results: Of the 1676 patients who received ethambutol for TB treatment, 987 had an ophthalmological symptom unrelated to EON, and 13 developed EON (incidence =0.28%). Demographic and clinical variables considered were age, gender, race/ethnicity, body mass index (BMI), and diabetes mellitus status. Examples of current infection information were chronic HIV and other comorbidities uniquely associated with renal function site/type treatment specific dose/duration ethambutol. Longer treatment duration significantly correlates with greater thickness of the left temporal retinal nerve fibre layer (RNFL) and thicker average RNFL. Increased peripapillary retinal nerve fibre layer (pRNFL) thickness on the left nasal RNFL scan was significantly correlated with ethambutol tablets (or count) (p=0.027).

Conclusion: The findings indicate that ethambutol toxicity is indeed dose and duration-dependent, evidenced by the relationship between prolonged treatment and increased RNFL thickness, suggesting heightened risk of visual impairment. Moreover, adverse effects were observed at even lower doses (12.3 mg/kg), reinforcing that no dose of ethambutol is entirely safe.

## Introduction

Tuberculosis (TB) has for some time been a worldwide general medical issue with both well-being and financial outcomes. India is one of the 30 countries with the most elevated predominance of TB and HIV/TB co-disease [1]. Ethambutol is a bacteriostatic anti-toxin used to treat Mycobacterium strains in mix with different prescriptions. It is related to the expanded endurance of tuberculosis patients. However, adverse effects, such as toxicity to the visual system, can harm quality of life and result in permanent disability [2].

Ethambutol is a bacteriostatic anti-toxin used to treat Mycobacterium strains in mix with different prescriptions. One of the most common and devastating side effects of ethambutol was optic neuropathy (EON). Patients with EON present with subacute visual disintegration, ordinarily beginning two to eight months after the inception of ethambutol [3]. Normal assessment discoveries are reciprocal visual sharpness decline, focal or centrocaecal visual field deformity, typical optic disc appearance, or gentle hyperemic enlarging in a beginning phase, developing to whiteness in a constant stage. The condition might bring about long-lasting visual disability, assuming that discontinuing the causative prescription is postponed [4].

The Revised National Tuberculosis Control Programme (RNTCP) was revamped in 2016 with a daily regimen to eliminate TB by 2025. The thrice-a-week regimen was changed to a daily regimen, and ethambutol has been made a part of both the intensive as well as continuation phases of the treatment [5-6].

Although this is expected to increase compliance and reduce drug resistance, there is an apprehension among ophthalmologists also observing in clinical settings that unless closely monitored, the incidence of optic neuropathy can increase multifold and result in significant visual impairment because of increased exposure to ethambutol.

The risk of eye-related side effects from ethambutol largely depends on how much and how long it’s taken. According to the World Health Organization, the recommended starting dose for treating *Mycobacterium tuberculosis *is between 15 and 20 mg/kg per day, where the chances of experiencing toxicity are about 1% to 3%. However, as the dose increases, so does the risk; at 25 mg/kg per day, the risk jumps to about 5% to 6%, and at 30 mg/kg per day, it can go as high as 18% to 30%. This risk is even greater for people with certain conditions, such as kidney issues, uncontrolled diabetes, hypertension, those over 65, and chronic smokers. It's crucial to remember that no dose is completely safe; there have even been reports of toxic effects at doses as low as 12.3 mg/kg [7].

This study aims to determine the incidence of ethambutol-induced optic neuropathy after the implementation of the Revised National Tuberculosis Control Programme (RNTCP) 2016 at a tertiary care center in Central India and to identify the risk factors associated with ethambutol-induced optic neuropathy (EON) as none of the studies are done in India on the incidence of EON post implementation of RNTCP.

## Materials and methods

This study was a retrospective observational study of patients who got ethambutol for TB treatment at the DOTS center of a tertiary care center in Bhopal after the implementation of revised NTCP 2016 guidelines. The Institutional Ethics Committee approved the study protocol (approval number IL0129). Although patient consent to review their medical records was not required, the confidentiality of patient identities and data has been protected.

Study subjects

Patients on a standardized treatment regimen for tuberculosis who received ethambutol at a tertiary care hospital in Bhopal, India, from January 2021 to September 2023 were identified through the hospital data. Subjects were grouped as those with ethambutol-induced optic neuropathy (EON) and those with no ethambutol-induced optic neuropathy (non-EON).

Inclusion criteria: Patients with confirmed EON during the study period as per international classification of disease -10 (ICD-10) code H47 (other disorders of the optic nerve and visual pathways) criteria were listed in the hospital data confirmed by chart review and by retrospective analysis of medical records of patients. Patients classified as controls (non-EON) were prescribed ethambutol during the research period but did not show signs of EON.

Variable definition

Demographic information, such as gender and age at the beginning of ethambutol treatment, was collected for each person. Details such as the primary site of tuberculosis, duration of anti-tubercular treatment, and the duration and dosage of ethambutol were recorded from subjects' medical records and hospital databases. Addiction history, such as cigarette smoking and alcohol consumption, was also recorded. Medical history was recorded, including the existence of any additional underlying illnesses such as diabetes, hypertension, and HIV infection. Ocular information such as color vision, visual acuity, and anomalies in the optic disc were taken from the medical files of EON participants. When accessible, the optical coherence tomography (OCT) results were documented.

Statistical analysis

The percentage of newly diagnosed instances of EON over the specified period compared to the total number of people treated with ethambutol for TB (EON + non-EON) was used to determine the EON incidence proportion, also known as cumulative incidence. The Fishers' Exact and Chi-square tests were used for categorical variables, and the t-test was used for independent samples to compare continuous variables to identify the components linked to EON. All factors that were different between groups and potentially could be associated with the development of EON were investigated by Logistic regression analysis. A p-value of less than 0.05 was considered statistically significant in our study. The data were analyzed using Statistical Package for the Social Sciences (SPSS) version 20.0 (IBM Corp., Armonk, NY).

## Results

Incidence of ethambutol-induced optic neuropathy (EON)

Thirteen (0.28%) of the 1676 patients who received ethambutol for TB treatment during the study period were diagnosed as EON. Of the 13 EON patients, five patients had primary pulmonary tuberculosis; two had tuberculosis of the cervical lymph node, two had tuberculosis of the hip, one had sternal Tuberculosis, one had Pott’s spine tuberculosis, one had tuberculosis mastitis, one had tuberculosis of skin (Table 1).

**Table 1:** Characteristics of patients treated with ethambutol with and without EON EON: Ethambutol-induced optic neuropathy; FDC: Fixed-dose combination. All values are represented in N (%) and ethambutol dose and duration in grams and months, respectively.

	Characteristic	Overall, N = 61^1^	Case, N = 13^1^	Control, N = 48^1^		
	Age (years)	45.33 (11.13)	48.08 (11.46)	44.58 (11.04)		
	Sex					
	Female	39 (63.93%)	9 (69.23%)	30 (62.50%)		
	Male	22 (36.07%)	4 (30.77%)	18 (37.50%)		
	BMI (kg/sqm)	24.46 (3.08)	23.88 (2.75)	24.61 (3.17)		
	BMI categories					
	Normal	37 (60.66%)	9 (69.23%)	28 (58.33%)		
	Overweight/obese	23 (37.70%)	4 (30.77%)	19 (39.58%)		
	Underweight	1 (1.64%)	0 (0.00%)	1 (2.08%)		
	Co morbidity					
	Absent	49 (80.33%)	9 (69.23%)	40 (83.33%)		
	Present	12 (19.67%)	4 (30.77%)	8 (16.67%)		
	Addiction history	8 (13.11%)	2 (15.38%)	6 (12.50%)		
	Type of Tuberculosis					
	Extra-pulmonary	25 (40.98%)	8 (61.54%)	17 (35.42%)		
	Pulmonary	36 (59.02%)	5 (38.46%)	31 (64.58%)		
HIV status						
Non-reactive		53 (86.89%)	11 (84.62%)		42 (87.50%)	
Reactive		8 (13.11%)	2 (15.38%)		6 (12.50%)	
Treatment duration (months)		8.33 (3.79)	9.54 (4.93)		8.00 (3.41)	
Ethambutol dose (g)		1,050.41 (106.60)	1,036.54 (120.60)		1,054.17 (103.57)	
Number of FDC tablets		3.82 (0.39)	3.77 (0.44)		3.83 (0.38)	

The ages of EON-affected patients ranged between 31 and 68 years (mean 48.08 ± 11.46 years); four were men, and nine were women. The average BMI was 23.88 ± 2.75 Kg/m^2^ (n=13). There were two (16%) patients with diabetes mellitus and one (8%) patient with hypertension. There were two HIV patients in the EON-affected group. Two (16%) patients had a history of cigarette smoking. Ethambutol's mean dose and duration were 1036 ± 120.60 g and 9.54 ± 4.93 months, respectively (Table 1).

Ophthalmic clinical characteristics

Visual Acuity

All patients with ethambutol-induced optic neuropathy had vision loss in both eyes. Visual acuity ranged from hand movement to 6/24 at presentation for retrospective analysis during this study. There was no statistically significant influence of ethambutol toxicity on measures of vision of the participants based on either the sex of the patient or the phase of treatment.

After stopping ethambutol, the visual function of only one patient (7.6%) improved by at least two lines on Snellen’s chart, with a mean recovery time of 9.4 months, while the remaining 12 patients showed no recovery. Patients who recovered did not show variations in demographics, past medical history, smoking habits, or ethambutol dosing compared to those who did not recover. Six patients who were able to take a color vision test were found to have a blue-yellow color deficiency. No improvement was seen in color vision deficiency in any of the participants.

Optical Coherence Tomography (OCT) Findings

Ophthalmic imaging was available in all 13 EON cases. The average RNFL thickness in the right eye and left eye was 113.54±3.07 microns and 119.21±2.67 microns, respectively. A statistically significant increase in average RNFL thickness, nasal and temporal RNFL thickness, was seen in contrast to the control group (p=0.045, p=0.036, and p=0.014). Three chronic patients showed pallor of the disc on OCT (Table 2).

**Table 2:** Thickness of RNFL in each quadrant measured on OCT Average Retinal Nerve Fiber Layer (RNFL) thickness was given eye-wise in microns as Mean +/-SD and p-value '< 0.05' was considered significant. OCT: Optical coherence tomography.

Parameters	Case (n=13)		Controls (n=48)		p-value
	Left Eye	Right Eye	Left Eye	Right Eye	
Average RNFL thickness (micron)	113.54±3.07	119.21±2.67	98.78±13.27	108.44±8.6	0.045*
Superior (micron)	81.30±15.19	80.91±26.76	72.98±78	84.97±21.98	0.021*
Inferior (micron)	73.07±11.56	71.98±12.39	69.75±10.76	77.89±11.78	0.88
Nasal (micron)	109.6±22.67	101.27±14.78	105.1±24.59	111.76±14.76	0.036*
Temporal (micron)	125.14±1.91	121.86±1.37	107.24±3.94	102.02±3.98	0.014*

Association of socio-demographic variables with ethambutol-induced optic neuropathy by unadjusted analysis

In unadjusted/univariate analysis, none of the predictors were found to predispose patients to developing ethambutol toxicity. There were no differences in age, gender, body mass index, hypertension, diabetes, HIV infection or site of tuberculosis, ethambutol dose and duration (Table 3).

**Table 3:** Association of socio-demographic variables with ethambutol induced optic neuropathy by unadjusted analysis In unadjusted/univariate analysis, none of the predictors were found to be significant. P-value '<0.05' was considered significant. OR = Odds Ratio, CI = Confidence Interval; FDC: Fixed-dose combination.

Characteristic	N	OR	95% CI	p-value
Age (years)	61	1.03	0.97, 1.09	0.3
Sex	61	—	—	—
Female	—	—	—	
Male	—	0.74	0.18, 2.64	0.7
BMI (kg/sqm)	61	0.92	0.75, 1.13	0.4
Treatment duration (months)	61	1.10	0.94, 1.27	0.2
Comorbidity	61	—	—	—
Absent	—	—	—	—
Present	—	2.22	0.51, 8.86	0.3
Addiction history	61	—	—	—
No	—	—	—	—
Yes	—	1.27	0.17, 6.47	0.8
HH status	61	—	—	—
Non-reactive	—	—	—	—
Reactive	—	1.27	0.17, 6.47	0.8
Ethambutol dose (g)	61	1.00	0.99, 1.00	0.6
Number of FDC tablets	61	0.67	0.16, 3.45	0.6
Type of Tuberculosis	61	—	—	—
Extra-pulmonary	—	—	—	—
Pulmonary	—	0.34	0.09, 1.19	0.10

Correlation between socio-demographic variables and measures of ocular examination by linear regression modeling

On correlating the RNFL thickness measurements with socio-demographic variables by linear regression modeling, we observed that an increased interval between treatment initiation and the appearance of visual symptoms is significantly associated with increased thicknesses of Left inferior RNFL and temporal RNFL. An increase in the duration of treatment is significantly associated with increased left temporal RNFL thickness and increased average RNFL thickness. An increase in left nasal RNFL thickness is significantly associated with ethambutol dose (or a number of tablets) (all the beta coefficients and p-values are mentioned in the table below). No such significant association was found for the right eye (Table 4).

**Table 4:** Correlation between socio-demographic variables and measures of ocular examination by linear regression modelling For all parameters, Beta coefficients and p-values are mentioned.  P-value '<0.05' was considered significant. CI = Confidence Interval; RNFL: Retinal nerve fibre layer; FDC: Fixed-dose combination.

Group	Characteristic	Beta	95% CI	p-value
Treatment initiation to visual symptom appearance duration (months)	Duration of treatment	0.41	0.19, 0.63	0.002*
Left Inferior RNFL Thickness	Treatment initiation to visual symptom appearance duration (months)	1.8	0.07, 3.4	0.042*
Left Temporal RNFL Thickness	Treatment initiation to visual symptom appearance duration (months)	2.2	0.42, 3.9	0.019*
Left Nasal RNFL Thickness	Ethambutol dose (g)	0.03	0.01, 0.05	0.021*
	Number of FDC tablets	8.0	1.5, 14	0.021*
Average Left RNFL thickness	Duration of treatment	0.33	0.05, 0.61	0.027*
	Duration of treatment	1.3	0.49, 2.1	0.005*

## Discussion

Ethambutol-induced optic neuropathy (EON) is one of the most prevalent and recognized forms of drug-induced optic neuropathy. Since ethambutol is a crucial part of many anti-mycobacterial treatment protocols, a significant number of tuberculosis patients worldwide may be affected by EON each year. One of the leading theories explaining EON centers on ethambutol's chelating properties, which interfere with lysosomal activation and disrupt oxidative phosphorylation, ultimately leading to neurotoxicity [8]. This interference causes calcium to flow into the mitochondria, inhibiting the electron transport chain and the production of adenosine triphosphate (ATP) [4]. Another key hypothesis suggests that the demyelination of the optic nerve, chiasma, and optic tract may contribute to this condition [9].

Incidences

Between 2012 and 2019, the incidence of ethambutol optic neuropathy at a tertiary care medical center in Thailand was 0.5% for all patients treated with ethambutol for tuberculosis and 1.88% for patients treated with ethambutol for tuberculosis with eye involvement [3]. By comparison, an incidence of 1.29% was identified in a study in Taiwan [10], whereas an incidence of approximately 1% was recorded in previous investigations [11]. In a recent study done in North India, the incidence of clinical EON was less than 2%, which corroborates our study, with most of the previous literature reporting the incidence in the 1-3% range [9]. In the current study, the overall incidence was 0.28%, which could be underestimated because hospital records are absent, the disease is undiagnosed, and there are other study limitations.

Risk factors

In unadjusted/univariate analysis, no correlation was found between EON and age, hypertension, smoking, ethambutol dosage, and duration. An investigation conducted on the Thai population revealed that advanced age was linked to EON, with comparable findings observed in previous studies [11-13]. Results in the current study are not comparable with a previous study conducted in a Taiwanese population by Chen et al., who found that most EON patients were older than 65 years [14]. In the current study, smoking and hypertension were not linked to EON. According to a study by Chen et al., hypertension and EON were related [14]. In the current study, smoking has not been linked to EON despite being a possible risk factor. Nonetheless, research has shown that smoking increases the chance of getting tuberculosis (TB), raises the risk of recurrent TB, and reduces the body's ability to respond to therapy [15]. Consequently, we speculate that smoking may raise the incidence of EON, which may be mediated by a more severe TB infection and may affect a patient's medication regimen. Ethambutol-associated optic neuropathy has been linked to patients with reduced renal function from renal TB, according to a number of studies. This could be because ethambutol is excreted by the kidneys [11-13]. However, in the current study, renal function was normal in all the patients with EON, so the analysis was not possible to find an association between renal function and EON.

Ocular findings

In our study, the patient presented with bilateral vision loss ranging from hand movement to 6/24, similar to previous studies. In ours, there was no significant improvement in visual acuity seen after discontinuation of ethambutol except in one patient, which could be due to delayed discontinuation of ethambutol, poor nutrition, delay in consultation, and negligence due to lack of awareness about EON as it is seen in developing countries [6]. A recent meta-analysis found a significant improvement in visual acuity when stopping EMB (P=0.035) [6]. Usually, it was discovered that after stopping ethambutol for one month, the course of EON was reversed [3]. Chamberlain et al. [16] showed that visual function recovery ranged from 40% to 80% in patients with ethambutol-induced optic neuropathy. A similar study in Thailand by Chaitanuwang et al. [3] showed visual function recovery in 60% of patients. However, few studies showed a sudden decrease in vision and persistent visual field defects even after discontinuing ethambutol and regular monitoring [17-19]. Three chronic patients in our study had disc pallor in the initial presentation, which may be a poor prognostic factor similar to the study by Chen et al. [14].

Out of 12 studies included in a meta-analysis on optic neuropathy in extended ethambutol usage, there was an initial reduction in color vision in eight studies, out of which four showed complete recovery, whereas four showed partial recovery. Overall, the analysis did not show a statistically significant improvement in color vision defect (P=0.181) [6]. In the present study also, none of the patients showed improvement in color vision after the stoppage of ethambutol.

Our study showed RNFL swelling with increased thickness in temporal and nasal quadrants. Similar findings were revealed by Chai et al. [20] and Kim et al. [21]. However, in a study by Zoumalant et al. [22], RNFL thickness was increased in the inferior quadrant. Many studies reported thinning of RNFL, particularly temporal RNFL, over a long time after ethambutol withdrawal. So, it can be considered that there is an increase in RNFL thickness initially due to axonal swelling due to ethambutol intake, and it is followed by a decrease in the thickness of RNFL over a long time of ethambutol withdrawal. In the current study, many patients were lost to follow-up, so it was not possible to measure the thickness of RNFL in long-term follow-up. Nevertheless, in some studies where they did follow up with OCT in EON patients, there was an improvement in OCT findings after stopping the usage of EMB [23-25]. Few studies failed to provide details regarding the final OCT outcomes upon discontinuation of ethambutol [8,21,26].

Dose and duration of ethambutol

The length and dosage of ethambutol were strongly correlated with the probability of EON, according to a prior meta-analysis [11]. The duration and dosage of ethambutol were longer in our study's EON participants; however, this correlation did not hold true in unadjusted models. This could be a result of the standardization of the recommended ethambutol dosage (15-25 mg/kg/d) to lower the risk of EON [3]. However, linear regression showed that the dose of ethambutol and duration of treatment with ethambutol had independent associations with increased average RNFL thickness and RNFL thickness in the left eye after adjustment for other covariates. Different studies showed that the incidence of ethambutol-induced ocular toxicity increased with increased dose, ranging from 1%-2.5% for a dosage of 15 mg/kg per day and increased to 5%-6% for a dosage of 25 mg/kg/day and reaching as high as 18% for dosage of 35 mg/kg per day [16,17,21,22,27]. However, a study by Choi et al. [28] showed that ethambutol-induced ocular toxicity could occur at low doses, such as 12.3 mg/kg per day. So, no dose of ethambutol can be considered a safe dose. The current study suggests that the total exposure to ethambutol is driven more by the duration of the standard dosing regimen rather than by higher daily doses.

Although the univariate analysis indicated no statistically significant differences in the likelihood of developing optic neuropathy based on critical factors such as age, gender, body mass index, presence of comorbidities, or specific ethambutol dosage and treatment duration, suggesting that these particular variables alone did not strongly predict EON in this cohort, the conclusion about the dose and duration dependence of ethambutol toxicity is supported by several key observations. While individual factors may not have shown significant effects, the cumulative data from various studies and the ethambutol drug pharmacodynamics suggest a consistent trend where higher exposure levels correlate with adverse outcomes. Even in our study, prolonged treatment duration is associated with increased RNFL thickness, which reflects toxicity.

Previous literature indicates that even established low doses (e.g., around 15 mg/kg) have reported toxicity incidences ranging from 1%-3%, which escalates with increased dosage to as high as 30% at higher doses (e.g., 30 mg/kg), which makes it evident that no dose is entirely devoid of risk [27]. The statement "no dose being safe" is validated by the fact that toxicity has been documented even at low dosages (12.3 mg/kg), further strengthening the understanding that risk for optic neuropathy exists regardless of the dosage administered.

Given the observed patterns of RNFL thickness and the documented toxicity in prior studies, healthcare providers need to routinely monitor patients on ethambutol therapy. This recommendation agrees with the conclusion that ethambutol carries inherent risks that entail vigilance, acknowledging that while individual factors in this study did not predict EON significantly, the potential for toxicity remains an ongoing concern [7,8]. It is important to note a few restrictions. First, selection bias is probably present because this was a retrospective study. Second, because our institution only had access to the computer-based diagnostic for the three years under study, our sample size was constrained. This study used imaging to rule out alternative causes, clinical diagnosis of ethambutol optic neuropathy, and observation that the patient's eyesight did not worsen after stopping ethambutol. Electrophysiological tests and perimetry were not conducted on any study subjects. Nevertheless, other potential risk factors leading to optic neuropathy can not be ruled out.

## Conclusions

The findings suggested that ethambutol's toxicity is relatively dose and duration-dependent, showed by the relationship between treatment and increased RNFL thickness, revealing an augmented risk of visual impairment. Moreover, adverse effects were observed at even lower doses (12.3 mg/kg), reinforcing that no dose of ethambutol is entirely safe. From our study, we can see despite the fixed dose and increased duration of ethambutol in RNTCP 2016, there was no significant increase in the incidence of EON after its implementation. So, apprehension of increased ocular toxicity due to an increased dose of ethambutol is very much ruled out. However, ethambutol can lead to irreversible visual loss, so the ophthalmic evaluation of patients on ethambutol should be routine practice in clinical settings, and such patients should be closely monitored. All the physicians who are prescribing ethambutol and the patients taking the ethambutol need to be made aware of the potential ocular toxicity of ethambutol. All physicians should counsel patients to immediately report if they notice any change in their vision after initiation of ethambutol. Ideally, a visual acuity chart and color vision chart should be there in the physician’s office to perform a quick vision assessment on every follow-up. Baseline ophthalmic examination should be a must before starting ethambutol in all patients, especially those with a high risk of developing EON (low weight, malnourishment, renal disease, coexisting diabetes mellitus). Additional research is needed to validate smoking as a potential risk factor, and it is essential to consider addressing this modifiable risk factor.
